# Dialogue between *Staphylococcus aureus* SA15 and *Lactococcus garvieae* strains experiencing oxidative stress

**DOI:** 10.1186/s12866-018-1340-3

**Published:** 2018-11-22

**Authors:** Clothilde Queiroux, Muriel Bonnet, Taous Saraoui, Pierre Delpech, Philippe Veisseire, Etienne Rifa, Cécile Moussard, Geneviève Gagne, Céline Delbès, Stéphanie Bornes

**Affiliations:** Université Clermont Auvergne, INRA, UMRF, F-15000 Aurillac, France

**Keywords:** *Lactococcus garvieae*, *Staphylococcus aureus*, Antimicrobial, Hydrogen peroxide, Gene expression

## Abstract

**Background:**

*Staphylococcus aureus* is an important foodborne pathogen. *Lactococcus garvieae* is a lactic acid bacterium found in dairy products; some of its strains are able to inhibit *S. aureus* growth by producing H_2_O_2_. Three strains of *L. garvieae* from different origins were tested for their ability to inhibit *S. aureus* SA15 growth. Two conditions were tested, one in which H_2_O_2_ was produced (high aeration) and another one in which it was not detected (low aeration). Several *S. aureus* genes related to stress, H_2_O_2_-response and virulence were examined in order to compare their level of expression depending on the inoculated *L. garvieae* strain. Simultaneous *L. garvieae* H_2_O_2_ metabolism gene expression was followed.

**Results:**

The results showed that under high aeration condition, *L. garvieae* strains producing H_2_O_2_ (N201 and CL-1183) inhibited *S. aureus* SA15 growth and impaired its ability to deal with hydrogen peroxide by repressing H_2_O_2_-degrading genes. *L. garvieae* strains induced overexpression of *S. aureus* stress-response genes while cell division genes and virulence genes were repressed. A catalase treatment partially or completely restored the SA15 growth. In addition, the H_2_O_2_ non-producing *L. garvieae* strain (Lg2) did not cause any growth inhibition. The SA15 stress-response genes were down-regulated and cell division genes expression was not affected. Under low aeration condition, while none of the strains tested exhibited H_2_O_2_-production, the 3 *L. garvieae* strains inhibited *S. aureus* SA15 growth, but to a lesser extent than under high aeration condition.

**Conclusion:**

Taken together, these results suggest a *L. garvieae* strain-specific anti-staphylococcal mechanism and an H_2_O_2_ involvement in at least two of the tested *L. garvieae* strains.

**Electronic supplementary material:**

The online version of this article (10.1186/s12866-018-1340-3) contains supplementary material, which is available to authorized users.

## Background

*Staphylococcus aureus* is an opportunistic human pathogen that can be responsible for food poisoning [1]. Its pathogenic activity is due to the production of various enzymes and toxins. It can be found in different environments including milk and dairy products [2]. In cheese, its level should not exceed 10^5^ CFU.g^− 1^ (European Community Regulation No. 852–853/2004).

*Lactococcus garvieae* is an ubiquitous LAB (Lactic Acid Bacteria) that can be found in various fermented foods including dairy products [3, 4], in fish, ruminant or human microbiota [5] and can be associated with pathologies such as fish lactococcoses [6, 7]. LABs such as *Lactococcus lactis* or *Lactococcus garvieae* are able to inhibit the proliferation of pathogens in cheese by the production of hydrogen peroxide [8], bacteriocins [9], by competition for nutrients [10, 11] or by acidification of the medium [12–14]. Depending on its concentration, hydrogen peroxide has a bactericide or a bacteriostatic effect on *S. aureus* [15]. *L. garvieae* raises our interest because a specific dairy strain of this LAB, N201 strain, can inhibit *S. aureus* growth by the production of H_2_O_2_ [2, 16, 17]. Moreover, it has a strong technological potential as a ferment for cheese production [18] and has almost no effect on acidification of the medium, compared to other LABs [2]. The inhibitive properties of *L. garvieae* N201 were confirmed on 2 strains of *S. aureus*: a human pathogenic strain, MW2 [19] and a non-pathogenic dairy strain, SA15, isolated from Saint-Nectaire cheese [17]. Delpech et al. [20] showed that *S. aureus* had no effect on *L. garvieae* growth and H_2_O_2_-related gene expression. On the contrary, *L. garvieae* N201 impaired the capacity of both strains of *S. aureus* to deal with the presence of H_2_O_2_ which led to growth deficiency [2, 16, 17].

In order to investigate the interaction between *S. aureus* and *L. garvieae* in oxidative stress-inducing culture conditions, genes involved in H_2_O_2_ metabolism were first choice targets, whose expression has been monitored by Delpech et al. [17, 20] in co-cultures of *L. garvieae* N201 and *S. aureus* SA15 or MW2.

In *L. garvieae*, superoxide dismutase *sod*A [21, 22] and pyruvate oxidase *pox*B genes [23, 24] are involved in H_2_O_2_ synthesis. As they do not have any catalase, LAB generally degrade H_2_O_2_ using alkyl hydroxyperoxidase (Ahp) [25] or glutathione peroxidase (Gpx) [26]. Thioredoxine reductase (Trx) are involved in response to Reactive Oxygen Species (ROS) [27, 28].

In *S. aureus*, Catalase (KatA) and Alkyl hydroxyperoxidase (Ahp) play a role in H_2_O_2_ degradation [29, 30]. Ahp leads to a dual function in oxidative-stress resistance, environmental persistence and host-pathogen interaction [29]. Amongst the targeted genes, *dna*K is known to be involved in H_2_O_2_-resistance [31, 32], *clp*C has an important role in oxidative stress regulation, and *cts*R is a transcriptional repressor of stress-genes [33]. Moreover, the 2 latter genes, belonging to the *dcw* cluster involved in cellular division, might be modulated by H_2_O_2_-stress, leading to *S. aureus* growth impairment [34]. So, if LAB can modify this genes cluster expression, it could have an inhibiting effect on cellular division.

In addition to *S. aureus* growth modulation, *L. garvieae* may also have an effect on its virulence. Enterotoxins are the main toxins responsible for *S. aureus* food poisoning. Ninety-four per cent of *S. aureus* isolated from cow milk have at least one enterotoxin-encoding gene [35]. Amongst the different enterotoxins, enterotoxin C, encoded by *sec*4 gene, is the most frequently involved in food poisoning [36–39]. Cretenet et al. [40] showed that this virulence-related gene expression can be modified by *L. lactis* in cheese. *S. aureus* virulence is under control of the *agr* system which is involved in regulating many stress response and virulence genes [41–43]. *agr*A is a response regulator [44] and is able to induce *hld*, a δ-lysin gene [43], and enterotoxin C encoding gene *sec*4 [45]. The *agr* system itself is controlled by several molecular intermediates such as SaeRS and SrrAB. SaeRS is a two-component system involved in response to environmental stress which could inhibit *agr* system [46] and also control virulence genes [47–50]. SrrAB is also a two-component system activated in an anaerobic environment [51]. It would be involved in virulence gene control [52] in response to H_2_O_2_. Indeed, in the presence of H_2_O_2_, *srr*A is repressed in *S. aureus* [53]. Under anaerobic conditions, SrrA represses *agr*A and *hld* expression [54, 55]. CodY, a regulatory protein involved in repressing virulence gene expression in *S. aureus,* is also involved in controlling *agr* system and virulence genes [56].

The level of aeration can change the level of H_2_O_2_-production according to the LAB strain. Indeed, it has been demonstrated that the transcriptomes of *L. lactis* and *L. garvieae* are significantly modified by the aeration level [20, 57]. *Lactobacillus crispatus* can produce H_2_O_2_ in high aeration conditions but not in static conditions [58]. Contrariwise, *Lactobacillus delbrueckii* subsp. *bulgaricus* can also produce H_2_O_2_ in static conditions, even though the amount produced is lower than in high aeration conditions [59].

The aim of the present study was to compare the transcriptional response involved in the antagonistic interaction between *S. aureus* and different strains of *L. garvieae*. In addition to N201 strain isolated from raw milk Saint-Nectaire cheese and already well described [2, 16], two other *L. garvieae* strains were selected for their different capacities to produce H_2_O_2_: a H_2_O_2_ -producing strain, CL-1183 (VIVASET, Veterinary Faculty, Complutense University from Madrid) isolated in Brazil from the milk from buffalo cows affected by subclinical mastitis [5] as well as a H_2_O_2_-non producing strain, Lg2, a fish pathogenic strain isolated in Japan [60]. The expression of *S. aureus* genes related to oxygen metabolism, response to stress, cell division and virulence was measured as well as the expression of *L. garvieae* genes related to oxygen metabolism.

## Results

In order to investigate the interaction between *S. aureus* and *L. garvieae* in oxidative stress-inducing culture conditions, we followed the growth of the strains and measured the amount of H_2_O_2_ in two culture conditions i.e. under high and low aeration levels. The genes whose expression has been monitored by Delpech et al. [17, 20] were chosen to compare the transcriptional response involved in the antagonistic interaction between *S. aureus* and different strains of *L. garvieae*. The expression of *S. aureus* genes related to oxygen metabolism, response to stress, cell division and virulence was measured as well as the expression of *L. garvieae* genes related to oxygen metabolism (Table 1).

**Table 1:** Differentially expressed *Staphylococcus aureus* genes in co-culture with 3 *Lactococcus garvieae* strains (N201, CJ-1183 and Lg2), summary table

	6 h			9 h			24 h		
Genes	N201	CL-1183	Lg2	N201	CL-1183	Lg2	N201	CL-1183	Lg2
High aeration									
Up-regulated	*–*	*dna*K	*–*	*clp*C *cst*R *dna*K *agr*A	*agr*A	*–*	*–*	*–*	*–*
Down-regulated	*sod*A *mra*W	*ahp*F *kat*A *mra*w *cod*Y	*–*	*kat*A *sod*A *mra*W *srr*A	*kat*A *mra*W *srr*A	*–*	*kat*A *sod*A *cod*Y *sae*S *sec*4	*ahp*F *sod*A *sec*4	*ahp*F *clp*C *dna*K *agr*A *cod*Y *hld*
Low aeration									
Up-regulated	*–*	*–*	*–*	*agr*A *sec*4	*ahp*F *agr*A *sec*4 *sel*2	*clp*C *cst*R *dna*K	*–*	*cod*Y	*agr*A
Down-regulated	*sec*4	*cod*Y	*–*	*–*	*–*	*cod*Y	*–*	*–*	*sod*A *srr*A

For detailed values, see Additional files 2 and 3

### Ability of *L. garvieae* strains to produce H_2_O_2_ and to inhibit *S. aureus* growth

**Fig. 1:**
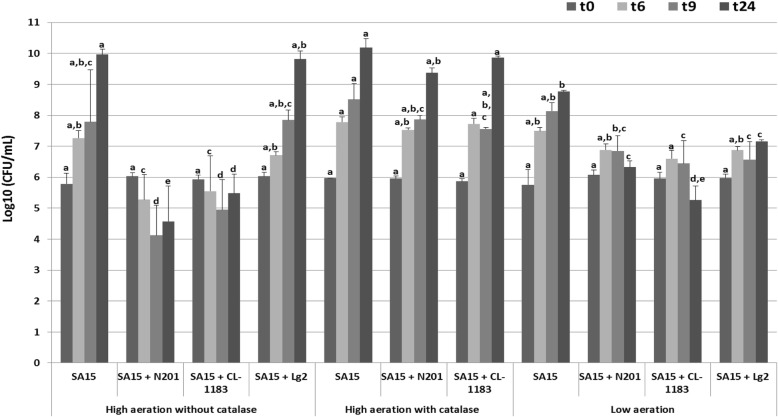
Effect of *Lactococcus garvieae* strains on *Staphylococcus aureus* SA15 growth, under high aeration levels (without and with catalase (4000 IU/ml)) and under low aeration level. ^a, b, c, d, e^ represent groups determined with LSD test, a same letter indicates values not significantly different (*p*-value < 0.05 by ANOVA1) through one Time

The effect of aeration level on microbial growth was tested by determining cellular concentrations of both microorganisms. In pure culture, *S. aureus* growth was almost identical under both high and low aeration conditions. However, the population count reached at 24 h was 1.2 log CFU/ml lower under low aeration than under high aeration (Fig. 1a). The 3 different strains of *L. garvieae* grew as well under high aeration level as under low aeration level independent of the presence of *S. aureus* (Additional file 1). pH values remained stable (7.0 ± 0.2) in all cultures over the whole experiment (data not shown).

Detectable amounts of H_2_O_2_ were produced exclusively under high aeration level by N201 and CL-1183 strains in co-culture with SA15. N201 strain produced from 1.327 mM ± 0.09 to 1.517 mM ± 0.18 of H_2_O_2_. The strain CL-1183 produced slightly more H_2_O_2_ than the strain N201, from 1.663 mM ± 0.23 to 2.415 mM ± 0.34. While N201 H_2_O_2_-production peak was at 9 h, CL-1183 H_2_O_2_-production stayed stable after 9 h (Table 2).

**Table 2:** H_2_O_2_ concentration produced by *Lactococcus garvieae* in pure culture and in co-culture with *Staphylococcus aureus* SA15, under high aeration

	Hydrogen peroxide concentration (mM)			
Time (h)	0	6	9	24
SA15	ND	ND	ND	ND
SA15 + N201	ND	1.343 ± 0.141	1.517 ± 0.180	1.327 ± 0.090
SA15 + CL-1183	ND	1.663 ± 0.230	2.415 ± 0.340	2.409 ± 0.240
SA15 + Lg2	ND	ND	ND	ND
N201	ND	1.455 ± 0.027	1.549 ± 0.040	1.105 ± 0.030
CL-1183	ND	1.257 ± 0.010	1.317 ± 0.030	1.148 ± 0.058
Lg2	ND	ND	ND	ND

*ND* not detected

Under high aeration, when co-cultivated with N201, SA15 growth was 2.1 log [CFU.ml^− 1^] lower than in pure culture as early as 6 h with a maximal growth inhibition of 5.8 log [CFU.ml^− 1^] at 24 h. SA15 growth co-cultivated with CL-1183 was 1.7 log [CFU.ml^− 1^] lower compared to pure culture as early as 6 h. The maximal growth inhibition was observed after 24 h of co-culture (4.5 log [CFU.ml^− 1^] lower). SA15 co-cultivated with Lg2 did not show any significant impairment in growth throughout the experiment compared to pure culture (Fig. 1a).

Under low aeration level and in co-culture with N201, CL-1183 or Lg2, SA15 growth was lower than in pure culture as early as 6 h (0.6 log [CFU.ml^− 1^], 0.9 log [CFU.ml^− 1^] or 0.6 log [CFU.ml^− 1^], with a maximal growth inhibition at 24 h (2.5 log [CFU.ml^− 1^], 3.5 log [CFU.ml^− 1^ or 1.6 log [CFU.ml^− 1^]], respectively) (Fig. 1a).

To determine the involvement of H_2_O_2_ produced by N201 and CL-1183 in growth inhibition of SA15, co-cultures of SA15 with these 2 strains were performed under high aeration level in presence of catalase (Fig. 1). No detectable amount of H_2_O_2_ was observed at any time. *S. aureus* cell concentration was not significantly different in presence of catalase compared to control (Fig. 1) during both exponential and stationary phases. Statistical analysis, using ANOVA, showed that in presence of catalase there was no significant difference of SA15 count after 24 h in co-culture with CL-1183 compared to pure culture. In contrary, SA15 cell concentration in co-culture SA15/N201 was still 0.8 log [CFU.ml^− 1^] lower than in pure culture. These results showed that the inhibition of SA15 growth by N201 and CL-1183 was partially or completely suppressed by catalase.

### Effect of *L. garvieae* strains on *S. aureus* genes expression under high aeration level (Additional file 2)

Whatever the *L. garvieae* strain cultivated with SA15, none of the H_2_O_2_-response genes tested was up-regulated. When SA15 was cultivated with *L. garvieae* N201 or CL-1183, *kat*A was down-regulated, at 9 h and 24 h with N201 (3.7 and 3.0-times, respectively), or at 6 and 9 h with CL-1183 (3.9 and 3.2-times, respectively). When cultivated with N201, *ahp*F expression in SA15 was not modified in comparison with pure culture, whereas in co-cultures SA15/CL-1183 and SA15/Lg2, it was down-regulated (2.1-times at 6 h and 2.9-times at 24 h, 6.9-times at 24 h, respectively). *sod*A was down-regulated at 6, 9 and 24 h in co-cultures with N201 (2.2, 4.4 and 3.9-times, respectively) or at 24 h (2.2-times) with CL-1183. No change in expression of *kat*A and *sod*A was observed with Lg2.

*L. garvieae* N201 induced an up-regulation of the three SA15 stress-response genes tested at 9 h; *clp*C, *cts*R and *dna*K were 2.0-times, 3.7-times and 2.5-times more expressed than in pure culture, respectively. When cultivated with CL-1183, only *dna*K was 2.8-times up-regulated at 6 h, while *clp*C and *cts*R expressions were not affected. Conversely, in co-culture SA15/Lg2, *clp*C and *dna*K were 3.9-times and 19.8-times down-regulated respectively, while *cts*R did not show any significant difference in its expression.

In both SA15/N201 and SA15/CL-1183 co-cultures, *mra*W cell division gene was strongly down-regulated at 6 and 9 h (7.0-times and 20.3-times, and 20.0-times and 12.0-times, respectively). In co-culture SA15/Lg2, *mra*W expression was not affected.

In co-culture, the five SA15 virulence-related regulator genes tested displayed contrasted patterns of expression. At 9 h, in both SA15/N201 and SA15/CL-1183 co-cultures, *agr*A was up-regulated (4.0-times with N201 and 3.4-times with CL-1183), while *srr*A was down-regulated (7.1-times or 6.4-times with N201 or CL-1183 respectively). *cod*Y expression was down-regulated 4.1-times at 24 h by N201 and 3.6-times at 6 h by CL-1183. *sae*S expression was 2.4-times lower at 24 h in co-culture with N201 than in pure culture, whereas it was not affected by CL-1183. *hld* expression remained stable until 24 h when SA15 was in co-culture with N201 or CL-1183. In co-culture SA15/Lg2, at 24 h, *agr*A, *cod*Y and *hld* expressions were all down-regulated (3.0-times, 2.6-times and 3.9-times, respectively), while *sae*S and *srr*A expressions remained stable.

In co-cultures SA15/N201 and SA15/CL-1183, amongst the 2 enterotoxins-encoding genes tested, only *sec*4 expression was modified at 24 h, with a 4.1-times and a 9.0-times down-regulation, respectively. *sel*2 expression was not modified whatever the SA15 culture conditions were. In co-culture SA15/Lg2, no enterotoxin gene expression was affected.

### Effect of *L. garvieae* strains on *S. aureus* genes expression under low aeration level (Additional file 3)

Under low aeration level, in presence of CL-1183, the H_2_O_2_-response gene *ahp*F was 4.2-times up-regulated at 9 h, whereas in presence of Lg2, *sod*A was 6.5-times down-regulated at 24 h. All the stress-response genes tested were up-regulated at 9 h (3.4-times for *clp*C, 4.0-times for *cts*R and 2.1-times for *dna*K) with Lg2. No change in stress-response genes expression was observed when SA15 was cultivated with N201 or CL-1183. Concerning the cell division gene *mra*W*,* its expression was not modified, whatever the strain cultivated with SA15.

Amongst virulence-related regulator genes, *agr*A was up-regulated in all three co-cultures at different stages of growth. It was the only virulence-related regulator gene differentially regulated by N201 (6.0-times up-regulated at 9 h). In the same way, this gene was 4.1-times up-regulated at 9 h by CL-1183, and 2.5-times up-regulated at 24 h by Lg2. *cod*Y was 2.0-times down-regulated at 6 h but 2.0-times up-regulated at 24 h with CL-1183, while *cod*Y and *srr*A were 2.1- and 2.7-times down-regulated at 9 and 24 h, respectively with Lg2.

The enterotoxin gene *sec4* was up-regulated in both N201/SA15 and CL-1183/SA15 co-cultures, (3.5- and 2.7-times, at 6 and 9 h respectively, with N201, and 2.5-times at 9 h with CL-1183). *sel*2 was 3.5-times up-regulated at 9 h only in the presence of CL-1183.

### Effect of aeration on *L. garvieae* strains (Additional file 4)

To evaluate the strain-specific effect of aeration on *L. garvieae,* expression of 5 H_2_O_2_-related genes of them (*trx*B1, *ahp*C, *gpx, pox*B and *sod*A) was monitored and compared between the 3 *L. garvieae* strains in co-culture with SA15. Overall, most differential expressions concerned H_2_O_2_-degradation genes. Differences between H_2_O_2_-producing and non-producing strains were essentially related to *ahp*C: both N201 and CL-1183 overexpressed this gene under low aeration (5.6-times at 24 h in N201, 4.0-times at 6 h and 6.5-times at 9 h in CL-1183). Conversely, Lg2 overexpressed *ahp*C (4.7-times) at 9 h under high level of aeration. Both CL-1183 and Lg2 overexpressed *trx*B1 under high level of aeration (2.9-times at 6 h in CL-1183 and 2.1-times 24 h in Lg2). Amongst H_2_O_2_-synthesis genes, only CL-1183 overexpressed *pox*B (4.1-times at 9 h under low aeration).

## Discussion

The aim of the study was to compare the transcriptional response involved in the antagonistic interaction between *S. aureus* and different strains of *L. garvieae*. Three strains of *L. garvieae* from different origins were used: N201, a dairy-isolated strain known to be a *S. aureus* inhibiting strain; CL-1183, a strain isolated from milk from buffalo cows suffering from mastitis; and Lg2, a fish pathogenic strain. Under high aeration condition, two of them were shown to be able to produce detectable amount of H_2_O_2_ (N201 and CL-1183), whereas the third one (Lg2) was not.

### *L. garvieae* strains and aeration level effect on *S. aureus* SA15 growth and on H_2_O_2_- and stress-responses

Under high aeration level, only *L. garvieae* N201 and CL-1183 inhibited *S. aureus* growth. CL-1183 down-regulated *S. aureus* H_2_O_2_-degradation genes (*ahp*F and *kat*A) and N201 and Lg2 only down-regulated *kat*A or *ahp*F respectively. This could mean that SA15 lost its ability to deal with H_2_O_2_-stress and suggest that the H_2_O_2_ detoxification occurred predominantly via KatA. These results are in accordance with those obtained by Cosgrove et al. [29], showing that *S. aureus ahp*C-*kat*A mutant was no more sensitive to H_2_O_2_ than the *kat*A mutant. They also found out that, Ahp would have less affinity for H_2_O_2_ than KatA and that it could be an alternative solution for H_2_O_2_-degradation when KatA was not functional. In our study, despite N201 only down-regulated *kat*A and not *ahp*F, AhpF did not play its compensatory role in H_2_O_2_ detoxification. This result can be explained by the fact that KatA was responsible for detoxifying high levels of H_2_O_2_, whereas AhpC was responsible for the removal of low levels of H_2_O_2_ [29]. This could be correlated with the large amounts of H_2_O_2_ detected in the co-cultures SA15/N201. The down-regulation of *kat*A, at 9 h and 24 h, can be explained by the fact that *srr*A was also down regulated. Mashruwala and Boyd [61] found that *srr*AB mutant strain had decreased transcription of genes encoding for H_2_O_2_ resistance factors as *kat*A. They also reported that SrrAB positively influenced H_2_O_2_ resistance during periods of high O_2_ dependent respiratory activity, but not when cellular respiration was diminished as a result of lower O_2_ availability. *S. aureus* SA15 genes involved in stress-response (*clp*C, *cts*R, *dna*K) were all up-regulated by N201 and only *dna*K was up-regulated by CL-1183, whereas *clp*C and *dna*K were down-regulated by Lg2. These results indicated that in presence N201 and CL-1183, SA15 detected an oxidative stress as a result of H_2_O_2_ production by these two *L. garvieae* strains. However, the up-regulation of these genes did not allow SA15 to fight against this stress, although precedent studies reported that *S. aureus clp*C or *dna*K mutants growth was impaired in the presence of H_2_O_2_ stress [32, 62, 63] and that a basic level of expression of *dna*K was sufficient in response to this stress [32]. All these data highlighted the complexity of the stress response machinery and the important role of *clp*C, *cts*R and *dna*K genes. Moreover, while *mra*W gene was not differentially expressed in presence of Lg2, it was repressed at 6 and 9 h by N201, confirming results obtained by Delpech et al. [17], and by CL-1183 in our study, when *S. aureus* growth was inhibited. The repression of *mra*W by N201 and CL-1183 was positively correlated with inhibition of SA15 growth. These results are supported by Cretenet et al. [40] showing that *L. lactis* was able to inhibit *fts*H, *fts*L and *fts*Z genes also involved in cellular division. Interestingly, several studies reported that inhibition of cell division protein is a promising approach for anti-staphylococcal therapy [64, 65].

Catalase treatment partially reduced SA15 growth inhibition by N201, confirming the result obtained by Delbes-Paus et al. [2], whereas the inhibition by CL-1183 was completely suppressed. Oogai et al. [66] observed that in presence of catalase, the *S. aureus* MW2 growth inhibition by *Streptococcus sanguinis* was completely suppressed. These observations confirmed the role of H_2_O_2_ produced by LAB in growth inhibition of *S. aureus*. Our study demonstrated that under high level of aeration, the inhibition of *S. aureus* by *L. garvieae* involved strain specific mechanisms. Indeed, CL-1183 inhibited SA15 growth mainly by H_2_O_2_ production whereas inhibition due to N201 may involve the combined action of H_2_O_2_ and other antagonistic mechanism.

Under low aeration condition, *S. aureus* growth was almost identical to that measured under high aeration condition, except after 24 h, when we observed a 1,2 log reduction in the *S. aureus* population level that could be due to the depletion of oxygen. Ledala et al. [67] showed that the growth rate of *S. aureus* was independent of oxygen limitation over 12 h although its metabolome was significantly affected. *S. aureus* growth was slightly inhibited by the three *L. garvieae* strains, although none of them could produce detectable amounts of H_2_O_2_ (Fig. 1). This suggests that there was another inhibitory mechanism involved. These results match those obtained with N201 in the previous studies [2, 17, 20]. Delbes-Paus et al. [2] have shown that in milk, even if H_2_O_2_ was not detected, *S. aureus* growth was inhibited by *L. garvieae* N201. However, no clear hypothesis to explain the mechanism of the anti-staphylococcal activity under low aeration could be drawn from the gene expression data. However, the up-regulation of stress-response genes in the presence of Lg2 was observed. Indeed, *clp*C, *cts*R and *dna*K genes, were all up-regulated at 6 h in co-culture with Lg2, suggesting that Lg2 triggered a stress on SA15 which led to a growth inhibition.

### *L. garvieae* strains and aeration level effect on *S. aureus* SA15 virulence gene expression

Under high level of aeration, *L. garvieae* N201 reduced *S. aureus* virulence-related genes expression confirming results obtained by Delpech et al. [17]. Cretenet et al. [40] and Queck et al. [68] have shown that the *agr* system, involved in the regulation of genes linked to *S. aureus* virulence, was repressed by *L. lactis* even if *L. lactis* does not produce H_2_O_2_ in this condition. Molecular intermediaries SaeRS and SrrAB are involved in controlling the *agr* system [46]. We showed *srrA* expression was repressed in co-culture with N201 and CL-1183, when H_2_O_2_ was produced as already observed by Chang et al. [53]. Moreover, Majerczyk et al. [56] have shown that a *cod*Y mutant could derepressed *agr* system. In our conditions, *cod*Y was repressed by CL-1183 at 6 h explaining *agr*A up-regulation at 9 h, whereas repression of *cod*Y at 24 h by Lg2 cannot explain *agr*A down-regulation suggesting that another regulator might be involved. Moreover, at 24 h, despite SA15 growth was not modified, Lg2 caused a down-regulation of three virulence-related regulator genes tested in this study (*agr*A, *cod*Y, *hld*). *sec*4 and *sel*2 are two enteroxin-encoding genes found in *S. aureus* SA15 [17]. Although enteroxin C-encoding gene *sec*4 is under the control of the *agr* system [45], *agr*A expression was only induced by N201 and CL-1183 and *sec*4 was repressed under high aeration level as shown by Delpech et al. [17]. Our data showed the repression of virulence associated genes and of enterotoxin encoding genes as well as simultaneous *S. aureus* growth inhibition in the presence of N201 or CL-1183, indicating that these 2 strains not only inhibited SA15 growth but also potentially attenuated its virulence.

Under low aeration condition, *S. aureus* over-expressed *agr*A and enterotoxin-encoding genes *sel*2 and *sec*4 in presence of N201 and CL-1183, whereas Lg2 did not induce any modification. In presence of Lg2, *agr*A was up-regulated at 24 h. This result can be explained by the fact that SA15 *srr*A gene was down-regulated, at the same time. The same observation was reported by Yarwood et al. [55]. They showed that transcription of RNAIII from the *agr* locus was inversely dependent on expression of *srr*AB. Inherently, despite SA15 *agr*A up-regulation by Lg2, no modification in enterotoxin genes expression was observed. Yarwood et al. [55] also reported that the SA15 *srr*B mutant growth was significantly slower in anaerobic condition, which is in accordance with SA15 growth inhibition in presence of Lg2.

### *L. garvieae* strain responses to low and high levels of aeration in co-culture with *S. aureus* SA15

It seemed that the three strains of *L. garvieae* differed essentially in their ability to degrade H_2_O_2_. Presence of H_2_O_2_ would not depend only on H_2_O_2_-synthesis, but also on H_2_O_2_-degradation by *L. garvieae* strains, which is an original mechanism, and in accordance with the results obtained by Delpech et al. [20].

In high aeration level, Lg2 overexpressed two H_2_O_2_ degradation genes *ahp*C and *trx*B at 9 and 24 h respectively. The non-detection of H_2_O_2_ could be explained by the absence of production of H_2_O_2_ or by its degradation by AhpC and TrxB. The latter mechanism could explain why, under low aeration, despite CL-1183 overexpressed an H_2_O_2_-synthesis gene, *poxB*, no detectable H_2_O_2_ was observed. Indeed, at the same time, this strain overexpressed the H_2_O_2_ degradation gene *ahp*C. Our data showed that the importance of the inhibition varies depending on the presence or absence of H_2_O_2_. Indeed, inhibition can occur while H_2_O_2_ is not detected. As regards other potential explanations for the growth inhibition, the only putative bacteriocin identified in the *L. garvieae* N201 genome was homologous to garvieaecin Q (GarQ, data not shown), a class IId bacteriocin [69]. In view of the results of previous experiments on *L. garvieae* N201 [20], the inhibition was probably caused neither by garvieaecin Q nor by a protein, nor by a lipid, and nor by a polysaccharide. In the present study, we evidenced *L. garvieae* strain-specific response in *S. aureus* gene expression which suggests that molecular mechanisms involved in the inhibition could differ between *L. garvieae* strains. Further investigations are needed to elucidate these mechanisms. A detailed analysis of strains physiology is needed to evaluate the potential role of nutritional competition in the inhibition. A global transcriptomic approach of the interaction would shed some light on the metabolic dialogue between these strains, and more particularly the potential involvement of quorum-sensing.

## Conclusions

Our data evidenced an impact of the aeration condition on the interaction between SA15 and *L. garvieae* strains.

Under high level of aeration, only N201 and CL-1183 produced detectable amounts of H_2_O_2_ and consequently inhibited SA15 growth. Both *L. garvieae* strains activated SA15 stress-response genes and inhibited the expression of several genes involved in H_2_O_2_-degradation, virulence and cellular division. A catalase treatment partially or completely suppressed this inhibition and no H_2_O_2_ was detected. Lg2 strain did not produce any detectable amount of H_2_O_2_ and did not inhibit SA15 growth. However, as N201 and CL-1183, Lg2 inhibited SA15 virulence-related genes and stress-response related genes.

Under low aeration level, none of the *L. garvieae* strains produced any detectable amount of H_2_O_2_. N201 and CL-1183 were still able to inhibit SA15 growth, as well as, Lg2. While N201 and CL-1183 induced the up-regulation of the SA15 enterotoxin-encoding genes and related regulator *agr*A, Lg2 caused up-regulation of SA15 stress-related genes and down-regulated virulence-related regulators genes.

The antagonistic properties of *L. garvieae* against *S. aureus* were strain- and aeration level-dependent. Under high aeration, they involved H_2_O_2_ production by two out of the three *L. garvieae* tested strains. This study provides new insights into microbial interactions mechanisms and shows the importance of investigating strain-specific effects.

## Methods

### Strains and culture conditions

Strains *Lactococcus garvieae* N201, CL-1183 and Lg2 and *Staphylococcus aureus* SA15 were cultivated in Brain-Heart Infusion buffered to pH 7 using Potassium Phosphate (BHI, Biokar Diagnostic, Pantin, France) at 30 °C and 37 °C, respectively, under static condition. After 20 h, cells were harvested at 3500 g during 15 min. Cell pellets were resuspended in 2 ml of BHI and syringed 5 times. Cell concentration was then determined using a Petroff-Hausser counting chamber. Fifty milliliters of buffered BHI, containing or not 4000 IU ml^− 1^ of catalase from bovine liver (ref. C100, Sigma), were then co-inoculated at ~ 10^7^ cells per ml for *L. garvieae* and ~ 10^6^ cells per ml for *S. aureus*, or just with *L. garvieae* or *S. aureus* at the same concentration in 250 ml Erlenmeyer flasks or in 50 ml Falcon tubes, as described previously [2, 17].

Then, Erlenmeyer flasks were incubated for 6, 9 or 24 h at 30 °C under shaking condition (150 rpm), corresponding to the high level of aeration condition. The low level of aeration condition corresponded to static fully filled 50 ml Falcon tubes. At 0, 6, 9 and 24 h, 1 ml of the cultures was removed to be syringed and serially diluted in Ringer’s solution. After adequate mixing, 100 μl of each dilution were plated onto solid Baird Parker for *S. aureus* and BHI agar for *L. garvieae* for numeration. Colony-forming units were counted after overnight incubation at 37 °C for *S. aureus* and 30 °C for *L. garvieae*. Also, 1 ml was removed from the cultures for H_2_O_2_ content analysis and 40 ml for RNA extraction. Cell pellets from 6, 9 and 24 h cultures were resuspended in 200 μl of cold Tris-EDTA buffer and then frozen at − 80 °C. The experiment was carried out in triplicate within each of three independent biological replicates, for a total of 9 samples per time point. pH measurements were made with the rest of the cultures.

### Quantitative analysis of H_2_O_2_ in *L. garvieae* cultures supernatant

Hydrogen peroxide concentration was determined according to Batdorj et al. protocol [70] with slight modifications. Cells were harvested by centrifugation at 11500 g, 15 °C for 10 min. One hundred microliter of the sample supernatant were mixed with 100 μl of 4-aminoantipyrine 4 mg.ml^− 1^ (Sigma-Aldrich, St. Louis, Missouri, USA), 20 μl of water-saturated phenol (Sigma-Aldrich, St. Louis, Missouri, USA), 750 μl of phosphate buffer Na_2_HPO_4_/NaH_2_PO_4_ 0.1 M (pH 7) and 30 μl of horseradish peroxidase type VI-A (500 U.ml^− 1^ in sodium phosphate buffer pH 6 (Sigma-Aldrich, St. Louis, Missouri, USA). Sample was mixed by inverting the tube and OD was measured at 505 nm. Blank was done by replacing sample by sterile medium. H_2_O_2_ concentration was determined using a standard curve performed with concentrations ranging from 0 to 3 mM. The minimal concentration that could be detected was 0.5 mM.

### RNA extraction and DNase treatments

Cells in Tris-EDTA buffer were thawed out and 25 μl of 20% SDS, 500 μl of phenol (pH 4), 3.5 μl of β-mercaptoethanol and 600 mg of Zirconium beads were added. The cells were broken twice in a tissue homogenizer (Precellys® 24, Bertin Technologies, Montigny-le-Bretonneux, France). Then, 200 μl of chloroform were added and mixed with the solution and sample was centrifuged at 11400 g for 20 min at 4 °C. RNA extraction was performed using NucleoSpin RNA Midi kit (Macherey-Nagel, GmbH & Co. KG, Düren, Germany) following the supplier’s instructions. The RNA extracts were treated twice with DNAse I using an Ambion DNA-free kit following the supplier’s instructions (Ambion, Inc., Austin, Texas, USA). The RNA extracts were quantified using a Nanodrop™ 2000C (Thermo Fisher Scientific Inc., Waltham, Massachusetts, USA).

### Reverse transcription and quantitative PCR

Reverse Transcription (RT) was performed using 0.5 μg of RNA twice treated with DNase, 5 μl of 10X buffer, 2 μl of 25X dNTP, 5 μl of random primers and 2.5 μl of retrotranscriptase (Applied Biosystems®, Life Technologies, Foster City, California, USA). The RT was done in a thermocycler (Techne® Prime, Bibby Scientific, Stone, Staffordshire, UK) with the following parameters: 10 min at 25 °C and 120 min at 37 °C.

Genes Of Interest (GOI) Ct were determined in quantitative PCR (qPCR) assays using 2.5 μl of cDNA suspensions 10-fold diluted in RNAse-free water and 10 μl of qPCR mix containing 1.25 μl of each primer (10 mM, Tables 3 and 4), 6.25 μl of qPCR Rotor Gene SybrGreen mix (Qiagen, Hilden, Germany) and 1.25 μl of RNAse-free water. The qPCR was performed according to the protocol on Rotor Gene Q (Qiagen, Hilden, Germany) with the following parameters: 4 min at 94 °C, then for 35 cycles, 30 s at 94 °C, 30 s at 55 °C, 60 s at 72 °C. Each reaction was performed in triplicate within each of three independent biological replicates, for a total of 9 reactions per time point.

**Table 3:** Targeted *Staphylococcus aureus* genes

Gene	Description	Category	Primers references
*hu* ^a^	DNA-binding protein	Housekeeping gene	[74]
*rec*A^a^	Recombinase A	Housekeeping gene	[74]
*agr*A	Accessory gene regulator A	Virulence-related regulator	[40]
*ahp*F	Alkyl hydroperoxidase F	H_2_O_2_-response	[17]
*clp*C	Clp proteinase C	Stress-response	[40]
*cts*R	Transcriptional repressor of stress-genes	Stress-response	[40]
*cod*Y	Transcriptional repressor	Virulence-related regulator	[17]
*dna*K	Chaperone protein DnaK	Stress-response	[40]
*hld*	Deltahemolysin	Virulence-related regulator	[40]
*kat*A	Catalase	H_2_O_2_-response	[40]
*mra*W	S-adenosyl methyltransferase	Cell division	[17]
*sae*S	Histidine protein kinase	Virulence-related regulator	[40]
*sec*4	Enterotoxin C	Enterotoxin	[40]
*sel*2	Enterotoxin L	Enterotoxin	[40]
*sod*A	Superoxide dismutase	H_2_O_2_-response	[40]
*srr*A	Staphylococcal respiratory response	Virulence-related regulator	[40]

^a^Reference genes

**Table 4:** Targeted *Lactococcus. garvieae* genes

Gene	Description	Category	Primers references
*tuf*B^a^	Elongation factor Tu	Housekeeping gene	[20]
*ahp*C	Alkyl hydroxyperoxide reductase	H_2_O_2_-degradation	[20]
*gpx*	Glutathione peroxidase	H_2_O_2_-degradation	[20]
*pox*B	Pyruvate oxidase	H_2_O_2_-synthesis	[20]
*sod*A	Superoxide dismutase	H_2_O_2_-synthesis	[20]
*trx*B1	Thioredoxin reductase	H_2_O_2_-degradation	[20]

^a^Reference gene

Primers efficiencies were determined according to a 10-fold template dilution standard and were all ranging from 1.90 (~ 95%) to 2.293 (~ 115%) according to the following equation: Efficiency = 10^(− 1/slope of calibration curve) [71]. The primers used were previously described by others [17]. One reference gene was used for *L. garvieae* data: *tuf*B encoding the elongation factor Tu. Considering that the lag in growth phase reflects the inhibition of *S. aureus* by *L. garvieae*, we evaluated gene expression at identical time points corresponding to different population levels. Two reference genes whose expression was stable over time under the different conditions were used for *S. aureus* data: housekeeping genes *rec*A, encoding the recombinase A, and *hu*, encoding a DNA-binding protein. The GOI expression was calculated according to the formula introduced by Hellemans et al. [72].

### Comparison of gene expression

Differential gene expression was evaluated by comparing expression between two conditions. First, the influence of aeration on gene expression in pure culture was evaluated for *S. aureus*. Then, the influence of different strains of *L. garvieae* (N201, CL-1183 and Lg2) on *S. aureus* SA15 gene expression was observed under high or low aeration condition. Secondly, *L. garvieae* gene expression was compared between the different strains in co-culture with *S. aureus* SA15 under both aeration conditions. A gene was considered as differentially expressed when its expression was changed by at least a factor of 2.

### Statistical analyses

Statistical analyses on microbial counts were performed using R software [73] by one-way analysis of variance (ANOVA) followed by Least Significant Difference (LSD) test. The gene expression statistical analyses were performed using R by ANOVA with Newmann-Keuls post-hoc test. Significance was declared at *P* < 0.05.

## Additional files

Additional file 1: **Figure. S2.** *L. garvieae* strains growth in pure culture and in coculture with *S. aureus SA15*, under different levels of aeration. (DOCX 116 kb)

Additional file 2: **Table S5.** Gene expression changes in *S. aureus* SA15 co culture with 3 L. garvieae strains under high aeration level. (DOCX 24 kb)

Additional file 3: **Table S6.** Gene expression changes in *S. aureus* SA15 co culture with 3 L. garvieae strains under low aeration level. (DOCX 24 kb)

Additional file 4: **Table S7.** Aeration induced changes in L. garvieae (N201, CL 1183 and Lg2 strains) H2O2-related gene expression in co-culture with *S. aureus* SA15. (DOCX 17 kb)

## Abbreviations

BHI: Brain heart infusion
GOI: Gene of interest
LAB: Lactic acid bacteria
ROS: Reactive oxygen species
