# Supplementary figures and images for: Dialogue between Staphylococcus aureus SA15 and Lactococcus garvieae strains experiencing oxidative stress
Source: BMC Microbiol. 2018 Nov 22;18:193. doi: 10.1186/s12866-018-1340-3 (PMC6251228; doi:10.1186/s12866-018-1340-3)

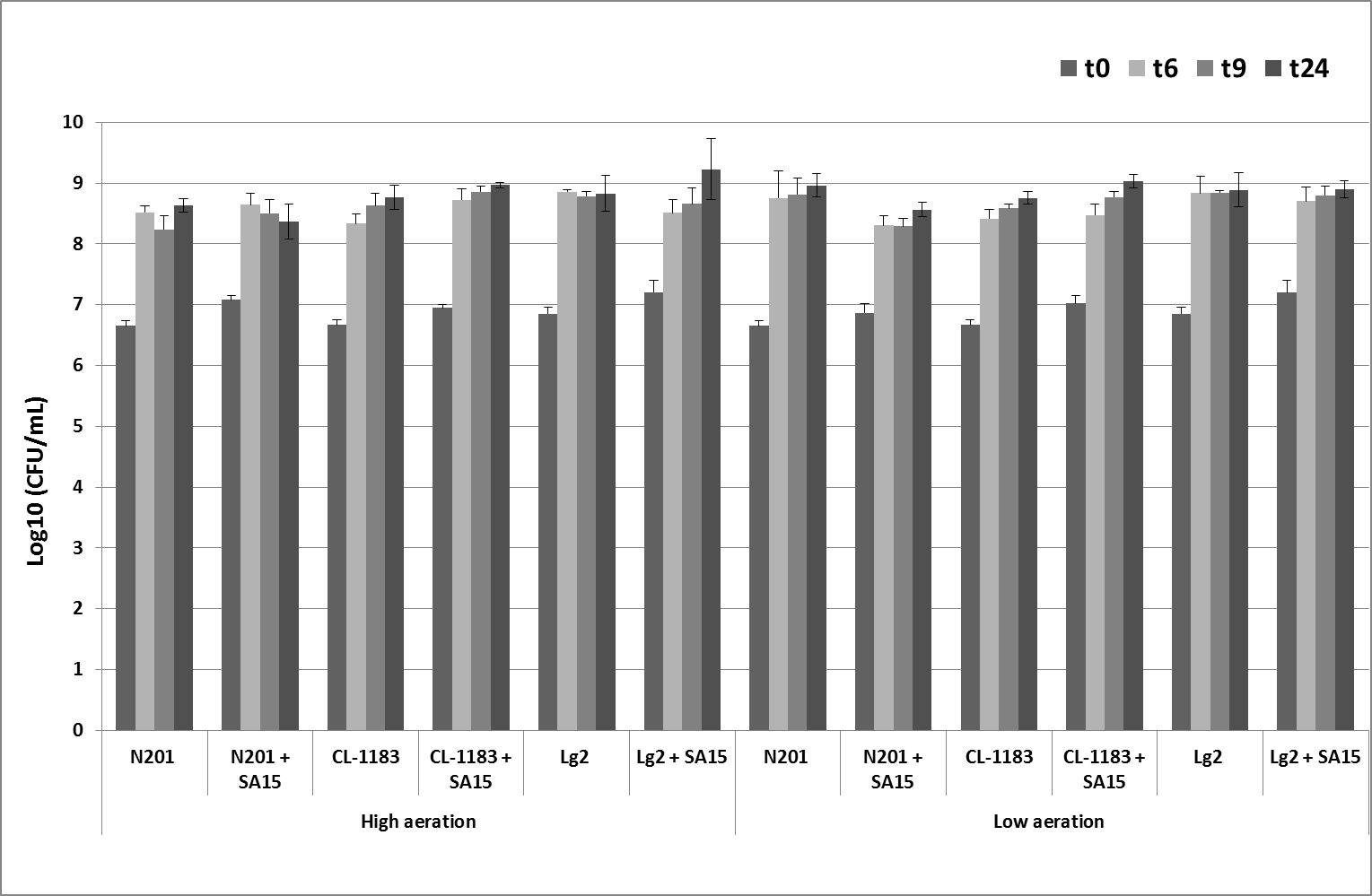

Supplement: Supplementary file 1 — Figure. S2. L. garvieae strains growth in pure culture and in coculture with S. aureus SA15, under different levels of aeration. (DOCX 116 kb) [file 12866_2018_1340_MOESM1_ESM.docx]
