# Supplementary material for: Dialogue between Staphylococcus aureus SA15 and Lactococcus garvieae strains experiencing oxidative stress
Source: BMC Microbiol. 2018 Nov 22;18:193. doi: 10.1186/s12866-018-1340-3 (PMC6251228; doi:10.1186/s12866-018-1340-3)
Supplement: Supplementary file 2 — Table S5. Gene expression changes in S. aureus SA15 co culture with 3 L. garvieae strains under high aeration level. (DOCX 24 kb) [file 12866_2018_1340_MOESM2_ESM.docx]

Table S5: Gene expression changes in *Staphylococcus aureus* SA15 in co-culture with 3 strains of *Lactococcus garvieae* under high aeration level

Genes were considered as differentially expressed when fold change was above 2 and when the p-value was lower than 0.1% (*), 0.05% (**), 0.01% (***) or 0.001% (****).

1. 6 h

|  | H_2_O_2_-response | | |  | Stress-response | | |  | Cell division |  | Virulence-related regulators | | | | |  | Enterotoxins | |
| --- | --- | --- | --- | --- | --- | --- | --- | --- | --- | --- | --- | --- | --- | --- | --- | --- | --- | --- |
| Genes up-regulated by *L. garvieae* | *ahp*F | *kat*A | *sod*A |  | *clpC* | *ctsR* | *dnaK* |  | *mraW* |  | *agrA* | *codY* | *hld* | *saeS* | *srrA* |  | *sec4* | *sel2* |
| N201 | 0.5 | 0.6 | 0.5 |  | 1.2 | 1.9 | 1.7 |  | 0.1 |  | 1.6 | 0.7 | 1.0 | 1.3 | 0.8 |  | 2.0 | 0.8 |
| 1183 | 0.5 | 0.3 | 0.8 |  | 1.0 | 1.8 | **2.8*** |  | 0.0 |  | 1.7 | 0.3 | 0.9 | 1.0 | 0.2 |  | 1.7 | 1.0 |
| Lg2 | 1.3 | 1.2 | 0.9 |  | 0.9 | 1.5 | 1.0 |  | 0.6 |  | 1.9 | 0.7 | 0.8 | 0.8 | 0.8 |  | 0.5 | 0.9 |

|  | H_2_O_2_-response | | |  | Stress-response | | |  | Cell division |  | Virulence-related regulators | | | | |  | Enterotoxins | |
| --- | --- | --- | --- | --- | --- | --- | --- | --- | --- | --- | --- | --- | --- | --- | --- | --- | --- | --- |
| Genes down-regulated by *L. garvieae* | *ahp*F | *kat*A | *sod*A |  | *clpC* | *ctsR* | *dnaK* |  | *mraW* |  | *agrA* | *codY* | *hld* | *saeS* | *srrA* |  | *sec4* | *sel2* |
| N201 | 1.9***** | 1.7 | 2.2* |  | 0.8 | 0.5 | 0.6 |  | **7.0*** |  | 0.6 | 1.5 | 1.0 | 0.7 | 1.3 |  | 0.5 | 1.3 |
| 1183 | **2.1**** | **3.9****** | 1.2 |  | 1.0 | 0.6 | 0.4 |  | **20.0**** |  | 0.6 | **3.6**** | 1.1 | 1.0 | 6.1 |  | 0.6 | 1.0 |
| Lg2 | 0.8 | 0.8 | 1.1 |  | 1.2 | 0.7 | 1.0 |  | 1.7 |  | 0.5 | 1.4 | 1.2 | 1.2 | 1.3 |  | 1.9 | 1.2 |

1. 9 h

|  | H_2_O_2_-response | | |  | Stress-response | | |  | Cell division |  | Virulence-related regulators | | | | |  | Enterotoxins | |
| --- | --- | --- | --- | --- | --- | --- | --- | --- | --- | --- | --- | --- | --- | --- | --- | --- | --- | --- |
| Genes up-regulated by *L. garvieae* | *ahp*F | *kat*A | *sod*A |  | *clpC* | *ctsR* | *dnaK* |  | *mraW* |  | *agrA* | *codY* | *hld* | *saeS* | *srrA* |  | *sec4* | *sel2* |
| N201 | 1.0 | 0.3 | 0.2 |  | **2.0*** | **3.7**** | **2.5*** |  | 0.0 |  | **4.0*** | 0.5 | 1.0 | 0.5 | 0.1 |  | 1.7 | 0.4 |
| 1183 | 0.6 | 0.3 | 0.2 |  | 1.3 | 1.7 | 1.6 |  | 0.1 |  | **3.4*** | 0.9 | 1.0 | 0.6 | 0.2 |  | 1.4 | 1.5 |
| Lg2 | 1.5 | 0.9 | 0.3 |  | 0.8 | 0.6 | 0.7 |  | 0.8 |  | 1.4 | 1.4 | 1.1 | 0.2 | 0.8 |  | 1.6 | 1.1 |

|  | H_2_O_2_-response | | |  | Stress-response | | |  | Cell division |  | Virulence-related regulators | | | | |  | Enterotoxins | |
| --- | --- | --- | --- | --- | --- | --- | --- | --- | --- | --- | --- | --- | --- | --- | --- | --- | --- | --- |
| Genes down-regulated by *L. garvieae* | *ahp*F | *kat*A | *sod*A |  | *clpC* | *ctsR* | *dnaK* |  | *mraW* |  | *agrA* | *codY* | *hld* | *saeS* | *srrA* |  | *sec4* | *sel2* |
| N201 | 1.0 | **3.7**** | **4.4*** |  | 0.5 | 0.5 | 0.4 |  | **20.3*** |  | 0.3 | 1.9 | 1.0 | 1.9 | **7.1**** |  | 0.6 | 2.7 |
| 1183 | 1.7 | **3.2*** | 5.7 |  | 0.8 | 0.6 | 0.6 |  | **12.0*** |  | 0.3 | 1.1 | 1.0 | 1.7 | **6.4**** |  | 0.7 | 0.7 |
| Lg2 | 0.7 | 0.7 | 3.7 |  | 1.3 | 1.8 | 1.3 |  | 1.3 |  | 0.7 | 0.7 | 0.9 | 0.8 | 1.2 |  | 0.6 | 0.9 |

1. 24 h

|  | H_2_O_2_-response | | |  | Stress-response | | |  | Cell division |  | Virulence-related regulators | | | | |  | Enterotoxins | |
| --- | --- | --- | --- | --- | --- | --- | --- | --- | --- | --- | --- | --- | --- | --- | --- | --- | --- | --- |
| Genes up-regulated by *L. garvieae* | *ahp*F | *kat*A | *sod*A |  | *clpC* | *ctsR* | *dnaK* |  | *mraW* |  | *agrA* | *codY* | *hld* | *saeS* | *srrA* |  | *sec4* | *sel2* |
| N201 | 0.5 | 0.3 | 0.3 |  | 0.7 | 1.0 | 1.0 |  | 0.2 |  | 1.2 | 0.2 | 0.3 | 0.4 | 0.5 |  | 0.2 | 1.6* |
| 1183 | 0.4 | 0.5 | 0.5 |  | 0.7 | 0.7 | 1.0 |  | 0.2 |  | 1.2 | 0.7 | 0.6 | 0.6 | 1.0 |  | 0.1 | 1.2 |
| Lg2 | 0.1 | 0.8 | 0.8 |  | 0.3 | 0.9 | 0.1 |  | 0.5 |  | 0.3 | 0.4 | 0.3 | 0.9 | 0.8 |  | 1.4 | 1.2 |

|  | H_2_O_2_-response | | |  | Stress-response | | |  | Cell division |  | Virulence-related regulators | | | | |  | Enterotoxins | |
| --- | --- | --- | --- | --- | --- | --- | --- | --- | --- | --- | --- | --- | --- | --- | --- | --- | --- | --- |
| Genes down-regulated by *L. garvieae* | *ahp*F | *kat*A | *sod*A |  | *clp*C | *cts*R | *dna*K |  | *mra*W |  | *agr*A | *cod*Y | *hld* | *sae*S | *srr*A |  | *sec*4 | *sel*2 |
| N201 | 2.1 | **3.0**** | **3.9***** |  | 1.5 | 1.5 | 1.0 |  | 4.6 |  | 0.8 | **4.1*** | 3.7 | **2.4*** | 1.9 |  | **4.1*** | 0.6 |
| 1183 | **2.9**** | 1.8***** | **2.2**** |  | 1.5 | 1.5 | 1.0 |  | 4.2 |  | 0.8 | 1.5 | 1.6 | 1.8 | 1.0 |  | **9.0*** | 0.8 |
| Lg2 | **6.9**** | 1.3 | 1.3 |  | **3.9**** | 1.1 | **19.8****** |  | 1.9 |  | **3.0*** | **2.6*** | **3.9**** | 1.1 | 1.2 |  | 0.7 | 0.9 |
