# Supplementary material for: Dialogue between Staphylococcus aureus SA15 and Lactococcus garvieae strains experiencing oxidative stress
Source: BMC Microbiol. 2018 Nov 22;18:193. doi: 10.1186/s12866-018-1340-3 (PMC6251228; doi:10.1186/s12866-018-1340-3)
Supplement: Supplementary file 3 — Table S6. Gene expression changes in S. aureus SA15 co culture with 3 L. garvieae strains under low aeration level. (DOCX 24 kb) [file 12866_2018_1340_MOESM3_ESM.docx]

Table S6. Gene expression changes in *Staphylococcus aureus* SA15 in co-culture with 3 strains of *Lactococcus garvieae* under low aeration level

Genes were considered as differentially expressed when fold change was above 2 and when the p-value was lower than 0.1% (*), 0.05% (**), 0.01% (***) or 0.001% (****).

1. 6 h

|  | H_2_O_2_-response | | |  | Stress-response | | |  | Cell division |  | Virulence-related regulators | | | | |  | Enterotoxins | |
| --- | --- | --- | --- | --- | --- | --- | --- | --- | --- | --- | --- | --- | --- | --- | --- | --- | --- | --- |
| Genes up-regulated by *L. garvieae* | *ahp*F | *kat*A | *sod*A |  | *clp*C | *cts*R | *dna*K |  | *mra*W |  | *agr*A | *cod*Y | *hld* | *sae*S | *srr*A |  | *sec*4 | *sel*2 |
| N201 | 1.2 | 0.8 | 0.6 |  | 0.9 | 1.4 | 1.4 |  | 0.6 |  | 1.3 | 0.8 | 1.3 | 1.0 | 1.3 |  | 3.5* | 0.8 |
| 1183 | 1.2 | 0.5 | 0.7 |  | 1.2 | 1.2 | 1.3 |  | 0.8 |  | 1.6 | 0.5 | 1.0 | 1.6 | 1.8 |  | 1.9 | 1.4 |
| Lg2 | 1.1 | 0.7 | 0.6 |  | 0.9 | 1.7 | 1.7 |  | 1.3 |  | 1.2 | 0.9 | 1.5 | 0.8 | 0.8 |  | 0.7 | 0.5 |

|  | H_2_O_2_-response | | |  | Stress-response | | |  | Cell division |  | Virulence-related regulators | | | | |  | Enterotoxins | |
| --- | --- | --- | --- | --- | --- | --- | --- | --- | --- | --- | --- | --- | --- | --- | --- | --- | --- | --- |
| Genes down-regulated by *L. garvieae* | *ahp*F | *kat*A | *sod*A |  | *clp*C | *cts*R | *dna*K |  | *mra*W |  | *agr*A | *cod*Y | *hld* | *sae*S | *srr*A |  | *sec*4 | *sel*2 |
| N201 | 0.9 | 1.3 | 1.6 |  | 1.1 | 0.7 | 0.7 |  | 1.7 |  | 0.8 | 1.3 | 0.8 | 1.0 | 0.8 |  | 0.3 | 1.2 |
| 1183 | 0.8 | 1.9* | 1.3 |  | 0.8 | 0.9 | 0.8 |  | 1.2 |  | 0.6 | 2.0* | 1.0 | 0.6 | 0.6 |  | 0.5 | 0.7 |
| Lg2 | 1.0 | 1.5 | 1.8 |  | 1.1 | 0.6 | 0.6 |  | 0.8 |  | 0.8 | 1.1 | 0.7 | 1.3 | 1.3 |  | 1.4 | 1.8 |

1. 9 h

|  | H_2_O_2_-response | | |  | Stress-response | | |  | Cell division |  | Virulence-related regulators | | | | |  | Enterotoxins | |
| --- | --- | --- | --- | --- | --- | --- | --- | --- | --- | --- | --- | --- | --- | --- | --- | --- | --- | --- |
| Genes up-regulated by *L. garvieae* | *ahp*F | *kat*A | *sod*A |  | *clp*C | *cts*R | *dna*K |  | *mra*W |  | *agr*A | *cod*Y | *hld* | *sae*S | *srr*A |  | *sec*4 | *sel*2 |
| N201 | 1.5 | 0.8 | 1.2 |  | 0.9 | 1.2 | 1.0 |  | 0.9 |  | 6.0** | 0.8 | 1.1 | 1.3 | 1.7 |  | 2.7* | 1.4 |
| 1183 | 4.2** | 1.4 | 1.0 |  | 1.1 | 1.2 | 1.5 |  | 0.7 |  | 4.1*** | 1.1 | 1.5 | 1.8 | 1.2 |  | 2.5** | 3.5*** |
| Lg2 | 1.6 | 0.8 | 0.8 |  | 3.4*** | 4.0*** | 2.1* |  | 1.3 |  | 1.3 | 0.5 | 0.9 | 0.7 | 1.4 |  | 0.3 | 1.1 |

|  | H_2_O_2_-response | | |  | Stress-response | | |  | Cell division |  | Virulence-related regulators | | | | |  | Enterotoxins | |
| --- | --- | --- | --- | --- | --- | --- | --- | --- | --- | --- | --- | --- | --- | --- | --- | --- | --- | --- |
| Genes down-regulated by *L. garvieae* | *ahp*F | *kat*A | *sod*A |  | *clp*C | *cts*R | *dna*K |  | *mra*W |  | *agr*A | *cod*Y | *hld* | *sae*S | *srr*A |  | *sec*4 | *sel*2 |
| N201 | 0.7 | 1.2 | 0.8 |  | 1.1 | 0.8 | 1.0 |  | 1.1 |  | 0.2 | 1.2 | 0.9 | 0.8 | 0.6 |  | 0.4 | 0.7 |
| 1183 | 0.2 | 0.7 | 1.0 |  | 0.9 | 0.8 | 0.7 |  | 1.4 |  | 0.2 | 0.9 | 0.7 | 0.6 | 0.8 |  | 0.4 | 0.3 |
| Lg2 | 0.6 | 1.3 | 1.2 |  | 0.3 | 0.2 | 0.5 |  | 0.8 |  | 0.8 | 2.1* | 1.1 | 1.5 | 0.7 |  | 3.1 | 0.9 |

1. 24 h

|  | H_2_O_2_-response | | |  | Stress-response | | |  | Cell division |  | Virulence-related regulators | | | | |  | Enterotoxins | |
| --- | --- | --- | --- | --- | --- | --- | --- | --- | --- | --- | --- | --- | --- | --- | --- | --- | --- | --- |
| Genes up-regulated by *L. garvieae* | *ahp*F | *kat*A | *sod*A |  | *clp*C | *cts*R | *dna*K |  | *mra*W |  | *agr*A | *cod*Y | *hld* | *sae*S | *srr*A |  | *sec*4 | *sel*2 |
| N201 | 1.6*** | 0.6 | 0.3 |  | 0.6 | 1.1 | 0.7 |  | 0.6 |  | 1.4* | 0.8 | 0.7 | 1.4 | 0.9 |  | 1.6 | 0.9 |
| 1183 | 1.3 | 0.1 | 0.1 |  | 0.7 | 0.6 | 0.7 |  | 1.1 |  | 1.4 | 2.0* | 0.3 | 1.6 | 1.3 |  | 1.1 | 2.0 |
| Lg2 | 0.8 | 0.1 | 0.2 |  | 0.8 | 0.9 | 0.9 |  | 1.5 |  | 2.5** | 0.7 | 0.8 | 0.9 | 0.4 |  | 1.5 | 1.1 |

|  | H_2_O_2_-response | | |  | Stress-response | | |  | Cell division |  | Virulence-related regulators | | | | |  | Enterotoxins | |
| --- | --- | --- | --- | --- | --- | --- | --- | --- | --- | --- | --- | --- | --- | --- | --- | --- | --- | --- |
| Genes down-regulated by *L. garvieae* | *ahp*F | *kat*A | *sod*A |  | *clp*C | *cts*R | *dna*K |  | *mra*W |  | *agr*A | *cod*Y | *hld* | *sae*S | *srr*A |  | *sec*4 | *sel*2 |
| N201 | 0.6 | 1.7 | 3.9 |  | 1.6 | 0.9 | 1.5 |  | 1.8 |  | 0.7 | 1.2 | 1.5 | 0.7 | 1.1 |  | 0.6 | 1.2 |
| 1183 | 0.8 | 14.0 | 16.3 |  | 1.5 | 1.8 | 1.4 |  | 0.9 |  | 0.7 | 0.5 | 3.1 | 0.6 | 0.8 |  | 0.9 | 0.5 |
| Lg2 | 1.2 | 11.7 | 6.5* |  | 1.2 | 1.2 | 1.1 |  | 0.7 |  | 0.4 | 1.5 | 1.2 | 1.1 | 2.7* |  | 0.7 | 0.9 |
