# Supplementary material for: Dialogue between Staphylococcus aureus SA15 and Lactococcus garvieae strains experiencing oxidative stress
Source: BMC Microbiol. 2018 Nov 22;18:193. doi: 10.1186/s12866-018-1340-3 (PMC6251228; doi:10.1186/s12866-018-1340-3)
Supplement: Supplementary file 4 — Table S7. Aeration induced changes in L. garvieae (N201, CL 1183 and Lg2 strains) H2O2-related gene expression in co-culture with S. aureus SA15. (DOCX 17 kb) [file 12866_2018_1340_MOESM4_ESM.docx]

Table S7. Aeration induced changes in *Lactococcus garvieae* (strains N201, Lg2 and 1183) genes expression in co-culture with *Staphylococcus aureus* SA15

Genes were considered differentially expressed when fold-change was above 2 and the p-value was lower than 0.1 (*), 0.05 (**), 0.01 (***).

Overexpression under high level of aeration

|  |  |  |  | N201 | | |  | 1183 | | |  | Lg2 | | |
| --- | --- | --- | --- | --- | --- | --- | --- | --- | --- | --- | --- | --- | --- | --- |
| Gene category |  | Gene |  | 6h | 9h | 24h |  | 6h | 9h | 24h |  | 6h | 9h | 24h |
| H_2_O_2_-degradation |  | *ahp*C |  | 0.7 | 0.7 | 0.2 |  | 0.3 | 0.2 | 0.7 |  | 1.0 | **4.7**** | 0.8 |
|  |  | *gpx* |  | 0.9 | 0.8 | 0.5 |  | 1.0 | 1.1 | 1.1 |  | 1.0 | 0.6 | 0.6 |
|  |  | *trx*B1 |  | 1.1 | 0.8 | 1.2 |  | **2.9**** | 1.0 | 1.3 |  | 1.2 | 0.8 | **2.1**** |
|  |  |  |  |  |  |  |  |  |  |  |  |  |  |  |
| H_2_O_2_-synthesis |  | *pox*B |  | 0.5 | 0.7 | 0.4 |  | 1.0 | 0.2 | 1.6 |  | 0.7 | 1.6 | 0.6 |
|  |  | *sod*A |  | 0.9 | 1.2 | 0.5 |  | 1.6 | 0.6 | 1.8 |  | 1.2 | 1.4 | 1.3 |

Overexpression under low level of aeration

|  |  |  |  | N201 | | |  | 1183 | | |  | Lg2 | | |
| --- | --- | --- | --- | --- | --- | --- | --- | --- | --- | --- | --- | --- | --- | --- |
| Gene category |  | Gene |  | 6h | 9h | 24h |  | 6h | 9h | 24h |  | 6h | 9h | 24h |
| H_2_O_2_-degradation |  | *ahp*C |  | 1.5 | 1.5 | **5.6*** |  | **4.0***** | **6.5***** | 1.4 |  | 1.0 | 0.2 | 1.2 |
|  |  | *gpx* |  | 1.1 | 1.3 | **2.0**** |  | 1.0 | 1.7 | 1.8***** |  | 1.0 | 0.9 | 0.9 |
|  |  | *trx*B1 |  | 0.9 | 1.2 | 0.8 |  | 0.3 | 1.0 | 0.8 |  | 0.8 | 1.2 | 0.5 |
|  |  |  |  |  |  |  |  |  |  |  |  |  |  |  |
| H_2_O_2_-synthesis |  | *pox*B |  | 1.9 | 1.4 | 2.6 |  | 1.0 | **4.1**** | 0.6 |  | 1.5 | 0.6 | 1.7 |
|  |  | *sod*A |  | 1.2 | 0.9 | 1.9****** |  | 0.6 | 1.8******* | 0.6 |  | 0.8 | 0.7 | 0.8 |
